# Intrahippocampally Injected Human Recombinant Clusterin Reduces Amyloid‐β Aggregate Size in Cerebral Arteriole Walls of Clusterin Knockout Mice

**DOI:** 10.1111/nan.70037

**Published:** 2025-09-08

**Authors:** Alexandru Laslo, Tudor‐Gabriel Boghițoiu, Laura Chinezu, Doina Manu, Adrian Dumitru Ivanescu, Bogdan Andrei Cordoș, Mark R. Wilson, Claudia Banescu, John D. Fryer, Ajay Verma, Jennifer M. Dewing, Roxana‐Octavia Carare

**Affiliations:** ^1^ Department of Urology George Emil Palade University of Medicine, Pharmacy, Science, and Technology of Târgu Mureș Târgu Mureș Romania; ^2^ Doctoral School of Medicine and Pharmacy George Emil Palade University of Medicine, Pharmacy, Science, and Technology of Târgu Mureș Târgu Mureș Romania; ^3^ Psychiatry Clinic 2 Mures County Clinical Hospital Târgu Mureș Romania; ^4^ Department of Histology George Emil Palade University of Medicine, Pharmacy, Science, and Technology of Târgu Mureș Târgu Mureș Romania; ^5^ Center for Advanced Medical and Pharmaceutical Research George Emil Palade University of Medicine, Pharmacy, Science and Technology of Târgu Mureș Târgu Mureș Romania; ^6^ Department of Anatomy George Emil Palade University of Medicine, Pharmacy, Science, and Technology of Târgu Mureș Târgu Mureș Romania; ^7^ Faculty of Medicine George Emil Palade University of Medicine, Pharmacy, Science, and Technology of Târgu Mureș Târgu Mureș Romania; ^8^ Centre for Experimental Medical and Imaging Studies George Emil Palade University of Medicine, Pharmacy, Science and Technology of Târgu Mureș Târgu Mureș Romania; ^9^ School of Science University of Wollongong Wollongong Australia; ^10^ Translational Genomics Research Institute Phoenix Arizona USA; ^11^ Foundry Engineering Boston Massachusetts USA; ^12^ Faculty of Medicine University of Southampton Southampton UK

**Keywords:** amyloid beta (Aβ), clusterin, confocal microscopy, intramural periarterial drainage

## Abstract

**Aims:**

The clusterin (*CLU*) gene is genetically associated with Alzheimer's disease (ad), and CLU levels have been shown to positively correlate with regional Aβ deposition in the brain, including in arteries from cerebral amyloid angiopathy (CAA) patients. CLU has also been shown to alter the aggregation, toxicity and blood–brain barrier transport of amyloid beta (Aβ) and has therefore been suggested to play a key role in regulating the balance between Aβ deposition and clearance in both the brain and cerebral blood vessels. However, it remains unclear whether the role of clusterin in relation to Aβ deposition is protective or pathogenic. The aim of this study was to determine how the presence of clusterin influences the pattern of Aβ deposition in hippocampal cerebral vessels.

**Methods:**

Intrahippocampal injections of fluorescent human recombinant Aβ alone or in combination with human recombinant CLU were carried out in *Clu* knockout mice. Aβ deposition and aggregate size in arterioles and capillaries were assessed by confocal microscopy.

**Results:**

The presence of CLU significantly reduced the size of Aβ deposits in the walls of cerebral arterioles but not in the tissue outside arterioles. There was no significant difference in overall Aβ deposition within cerebral arterioles and capillaries of mice injected with Aβ + CLU versus Aβ alone.

**Conclusions:**

Our findings confirm that CLU directly impacts cerebral vascular Aβ aggregation, the implications of which are particularly relevant to CAA, which is a major cause of cerebral haemorrhage and cognitive decline, particularly in individuals with ad.

## Summary

- Intrahippocampal injection of human recombinant clusterin (CLU) alone or in combination with fluorescently tagged amyloid beta (Aβ) was carried out in clusterin knockout mice
- Co‐injection of CLU with Aβ significantly reduced the size of Aβ deposits in cerebral arteriole walls
- The presence of CLU did not significantly change the total amount of Aβ deposited in cerebral arterioles and capillaries

## Graphical abstract

This study examined how clusterin influences amyloid beta (Aβ) deposition in the brain's blood vessels using clusterin deficient mice. The presence of human recombinant CLU, introduced by intrahippocampal injection, reduced the size of Aβ deposits in arteriolar walls but did not affect overall Aβ levels. These results suggest CLU plays a direct role in modulating vascular Aβ aggregation, with implications for understanding cerebral amyloid angiopathy in Alzheimer's disease.

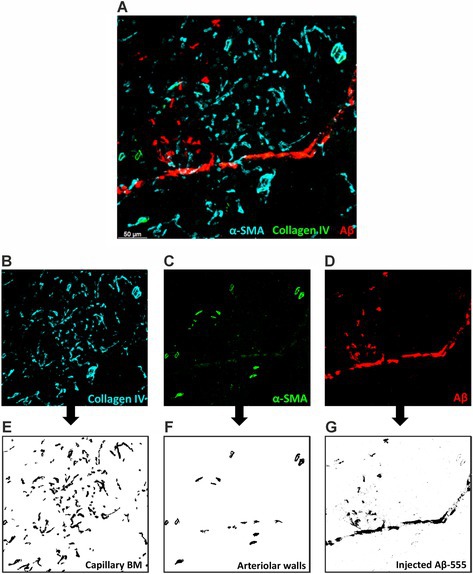

**Abbreviations**
ad
: Alzheimer's disease
AF: arbitrary fluorescence
Aβ: amyloid beta
BBB: blood brain barrier
CAA: cerebral amyloid angiopathy
IPad
: intramural periarterial drainage
PBS: phosphate buffered saline

## 1 Introduction

Alzheimer's disease (ad) is the most common form of age‐related dementia and represents a major health problem in the growing population of elderly people in developed countries [1]. One key element of the neuropathology of AD is the deposition of amyloid beta (Aβ) within the cerebral blood vessel walls, as cerebral amyloid angiopathy (CAA), mainly in cortical arterioles and leptomeningeal arteries [2, 3, 4, 5]. Clinical and experimental data demonstrate that the failure of clearance of Aβ from the brain is an essential element of the pathogenesis of AD, CAA and of complications after immunisation against Aβ [6, 7]. A major pathway by which Aβ drains from the brain to cervical lymph nodes is along the basement membranes of the vascular smooth muscle cells of the walls of cerebral arteries, in the direction counter to blood flow, referred to as Intramural Peri‐Arterial Drainage (IPAD) pathways [8]. The failure of clearance of Aβ from the extracellular spaces of the brain along these pathways is a key pathogenic factor in the development of CAA [9]. Ageing, possession of *APOE4* genotype, immunisation against Aβ and a high fat diet all result in changes in the structure of the IPAD pathways of Aβ [10, 11, 12, 13]. In addition to IPAD pathways, Aβ is also cleared from the brain via low density lipoprotein‐related protein 1 (LRP1) and via glymphatic clearance [6].

Clusterin (Apolipoprotein J or ApoJ) is a disulphide linked heterodimeric glycoprotein. Genome‐wide association studies of sporadic Alzheimer's disease, in which Aβ accumulates as plaques in the cerebral cortex, as well as in deep grey nuclei such as the basal ganglia, brainstem nuclei and cerebellar cortex and within vessels as CAA, have highlighted the importance of common genetic variations in the gene encoding clusterin [14]. Experimental work suggests that clusterin plays a major role in the clearance of Aβ42 via low density lipoprotein‐related protein 2 (LRP2) [15]. Neuropathology studies using postmortem human brains showed that clusterin appears to be sequestered with Aβ species in sporadic CAA [16, 17, 18]. Although the predominant species of Aβ in CAA is Aβ40, with progressive failure of IPAD of interstitial fluid, there is also accumulation of Aβ42 in the walls of blood vessels, and there is a significant positive correlation between clusterin concentration and regional levels of insoluble Aβ42 in human brains [19]. Our own data using leptomeningeal arteries from human brains demonstrated an upregulation of clusterin in CAA arteries, possibly due to either entrapment of the Aβ‐clusterin complex in the perivascular drainage pathways or a compensatory upregulation of clusterin to clear the excess Aβ42 that cannot be eliminated normally [20].

The role of CLU in parenchymal Aβ pathology relates to its ability to influence the aggregation state of Aβ and its fibril formation. Whereas differences in Aβ species, aggregation protocols and incubation strategies have produced variable results, a common finding across multiple studies is that clusterin is able to bind to Aβ oligomers of varying sizes and interfere with Aβ aggregation to prevent fibril formation [21]. Whereas studies investigating how this translates to amyloid toxicity have remained contradictory [21, 22, 23, 24, 25], clusterin has been shown to prevent the toxicity of Aβ42 oligomers in glial cells and neurons, as well as improving memory in a rodent model [26]. Using highly sensitive single‐molecule fluorescence methods, it has been shown that specifically the oligomeric soluble forms of Aβ40 interact with clusterin to form stable complexes [27]. It is difficult to synthesise pure recombinant clusterin, as the experimental conditions result in the simultaneous formation of misfolded proteins in the media, attaching to the clusterin and interfering with its biological properties [28, 29].

When APP/PS1 mice were bred onto a *Clu* ^*+/+*^ or a *Clu* ^*−/−*^ background, the APP/PS1/*Clu* ^*+/+*^ mice showed Aβ deposition mostly in the form of parenchymal plaques, whereas in APP/PS1/*Clu* ^*−/−*^ mice, Aβ was predominantly deposited in the cerebral vasculature as CAA [30]. This suggests that CLU is critical for the effective clearance of Aβ along the brain vasculature. The deposition of Aβ in the walls of arteries, but not veins, in APP/PS1/*Clu* ^*−/−*^ mice supports the continued investigation of the role of CLU facilitating Aβ clearance in the IPAD system. We hypothesise that the administration of recombinant human CLU in transgenic *Clu* knockout mice will significantly reduce human recombinant Aβ accumulation within the IPAD system and alter its distribution pattern and thus facilitation of IPAD.

## 2 Materials and Methods

### 2.1 Animals and Procedures

The study was conducted at the George Emil Palade University of Medicine, Pharmacy, Science and Technology of Targu Mures, Romania. All the procedures on mice were approved by the Veterinary Health and Food Safety Institution of Romania and in compliance with the guidelines established by the Public Health Service Guide for the Care and Use of Laboratory Animals (Ethical approval 59/2023).

Six‐week‐old *Clu* −/− (clusterin knockout) mice bred on C57Bl6/J background strain (*n* = 10, male and female) were obtained from the Mayo Clinic (United States) with the assistance of Dr. John Fryer. The genotype for the *Clu* −/− mice was determined using the PureLink Genomic DNA Mini Kit (ThermoFisher Scientific, United States), with the following primers: Clu_Fw_c_CGC TAT AAA TAG GGC GCT TC; Clu_Rev_w_ TGA TGG GGC TCT AGT CAC CT; and Clu_Rev_m_ GCC AGA GGC CAC TTG TGT AG.

Mice were anaesthetised with isoflurane mixed with concentrated O_2_ (1.7 L/min). They were induced with 5% isoflurane for 5 min and then maintained with 2.5% isoflurane for 5 min. The mice were positioned on a heated pad on the stereotaxic frame using ear bars and a mouthpiece to secure the head and prevent movement when pressure was applied to the scalp. The 10 *Clu* −/− mice were split into two groups of *n* = 5. Intrahippocampal injection of Aβ (human amyloid beta [1, 2, 3, 4, 5, 6, 7, 8, 9, 10, 11, 12, 13, 14, 15, 16, 17, 18, 19, 20, 21, 22, 23, 24, 25, 26, 27, 28, 29, 30, 31, 32, 33, 34, 35, 36, 37, 38, 39], HiLyteTM Fluor 555—labelled Eurogentec, Cat. No. AS‐60492) was carried out in group 1 (Aβ only) (*n* = 5, 3 male and 2 female), and in combination with human recombinant CLU (Professor Mark Wilson, the University of Wollongong, Australia, R&D Systems, Cat. No. 2937‐HS) in Group 2 (Aβ + CLU) (*n* = 5, 3 male and 2 female) (Aβ + CLU). Prior to the injection, Aβ was mixed on ice in a 10:1 ratio with clusterin and injected into the mice immediately thereafter.

After exposing the skull through a median incision, the surface was cleaned using phosphate buffered saline (PBS, pH 7.4, ThermoFisher, cat. No. 70011044) and cotton buds. The target was chosen by calibrating the axis of the stereotaxic machine with the mice. Injection in the hippocampal area was performed using a Hamilton syringe with a 33‐gauge Hamilton needle (Essex Scientific Laboratory Supplies Ltd.) at a rate of 0.5 μL over 2.5 min, as before [31]. This technique allows for the injection of small amounts of substances into the brain parenchyma at a controlled and physiologically relevant rate. The recombinant human clusterin bearing a C‐terminal C‐tag was expressed in MEXi293E cells (IBA Life Sciences) and purified using a Capture Select C‐tag column (Thermo Fisher Scientific, Australia), as described in [32]. After 10 min, terminally anaesthetised mice were intracardially perfused with PBS, followed by 4% paraformaldehyde (Silver Chemicals, cat. No. STF398) at a rate of 5 mL/1 min. Based on our previous studies, this 10‐min postinjection time point is optimal for the analysis of IPAD, ensuring detection of intramural Aβ prior to it being cleared from the brain [33, 34, 35].

The brains were dissected from the skull and fixed in 4% paraformaldehyde for 3 h and then transferred into 30% sucrose for 6 h. The cerebellum and brain stem were separated from the cerebral hemispheres. The brain was placed into a base mould with the frontal lobes facing upwards and covered with OCT cryo‐embedding media (VWR, 361603E). Tissue was stored at −8°C until ready to section. Coronal slices of CA1 hippocampus, 20‐μm thick, were sectioned using a Leica CM1860 UV cryostat. The sections were mounted on SuperFrost slides (SuperFrost PlusTM adhesion slides, Thermo ScientificTM, 10149870) and visualised using a Zeiss Axioskop 2 equipped with a rhodamine filter to identify the section containing the injection site. Previous studies in mice have observed Aβ [1, 2, 3, 4, 5, 6, 7, 8, 9, 10, 11, 12, 13, 14, 15, 16, 17, 18, 19, 20, 21, 22, 23, 24, 25, 26, 27, 28, 29, 30, 31, 32, 33, 34, 35, 36, 37, 38, 39] drainage occurs predominantly in the posterior direction and can be visualised in the wall of blood vessels up to 200 μm from the injection site. Therefore, we chose coronal sections 200‐μm posterior to the injection site for immunohistochemistry. All sections were stored at −20°C.

### 2.2 Immunofluorescence and Confocal Imaging

To remove any remaining OCT, the slides were washed with 0.01‐M PBS three times for 5 min and then blocked with 15% goat serum (Sigma Aldrich, cat. No. G9023) for 45 min at room temperature. The hippocampal brain sections were then incubated overnight in a humid chamber at 4°C with rabbit anticollagen IV antibody (Col IV, dilution 1:400, Abcam, cat. No. ab6586) and anti‐α‐Smooth Muscle Actin‐FITC (SMA, dilution 1:200, Sigma Aldrich, cat. No. F3777). The sections were then washed with 0.01‐M PBS three times for 10 min and incubated with secondary antibody (conjugated secondary antibody goat antirabbit AlexaFluor 633, dilution 1:200, ThermoFisher Scientific, A‐21070) for 1 h at room temperature. The sections were then washed with 0.01 M PBS three times for 10 min and mounted using a fluoroshield mounting medium with DAPI (Abcam, cat. No. ab104139‐20) and stored at 4°C until image analysis.

For each section, images from the left hippocampus were captured using a Leica TCS SP8 confocal microscope. Acquisitions were made with a dry HC PL APO 20×/NA = 0.75 objective, with an optical zoom of 1. The emitted signals came from an area of 581.25 μm × 581.25 μm on Hybrid detectors (HyD) or photomultiplier tube (PMT). Confocal sequential imaging was used to prevent fluorochrome emission overlap. The matrix size was 1024 × 1024 pixels.

### 2.3 Image Processing

Image processing was performed using ImageJ (v1.54i) with the MorphoLibJ (v1.6.2) and Bio‐Formats (v7.3.0) plugins (https://imagej.nih.gov/ij, accessed in March 2024). A custom macro was developed to generate maximum Z‐projection images and identify capillaries and arterioles.

To identify arterioles, we selected structures positive for both Collagen IV (marking all vessel basement membranes) and a complete ring of α‐SMA (marking vascular smooth muscle cells). To establish a robust minimum size threshold, a calculation was performed based on the anatomical definition of an arteriole (minimum 10 μm diameter) and the study's image calibration (0.5676 μm/pixel). The theoretical minimum cross‐sectional area of the stained arteriolar wall—excluding the nonstained lumen—was calculated to be approximately 155 pixels. Based on this, a minimum area threshold of 150 pixels (approximately 48.3 μm^2^) was applied using the ‘Analyse Particles’ function. Objects positive for both stains but smaller than this threshold were excluded from the arteriole classification.

To define capillaries, a comprehensive mask of all Collagen IV‐positive vasculature was first generated. The previously identified arteriole mask was then dilated by 4 pixels (to avoid edge effects) and subtracted from this comprehensive Collagen IV mask, isolating the remaining nonarteriolar vessels. A minimum area threshold for these capillary structures was determined based on the cross‐sectional area of a typical small capillary (3.5‐μm diameter). Given the image calibration, this corresponds to a calculated area of approximately 29.2 pixels^2^. Therefore, a threshold of 29 pixels (approximately 9.6 μm^2^) was applied to the remaining Collagen IV‐positive structures to define them as capillaries. Objects smaller than this threshold were excluded from the final analysis.

**FIGURE 1:**
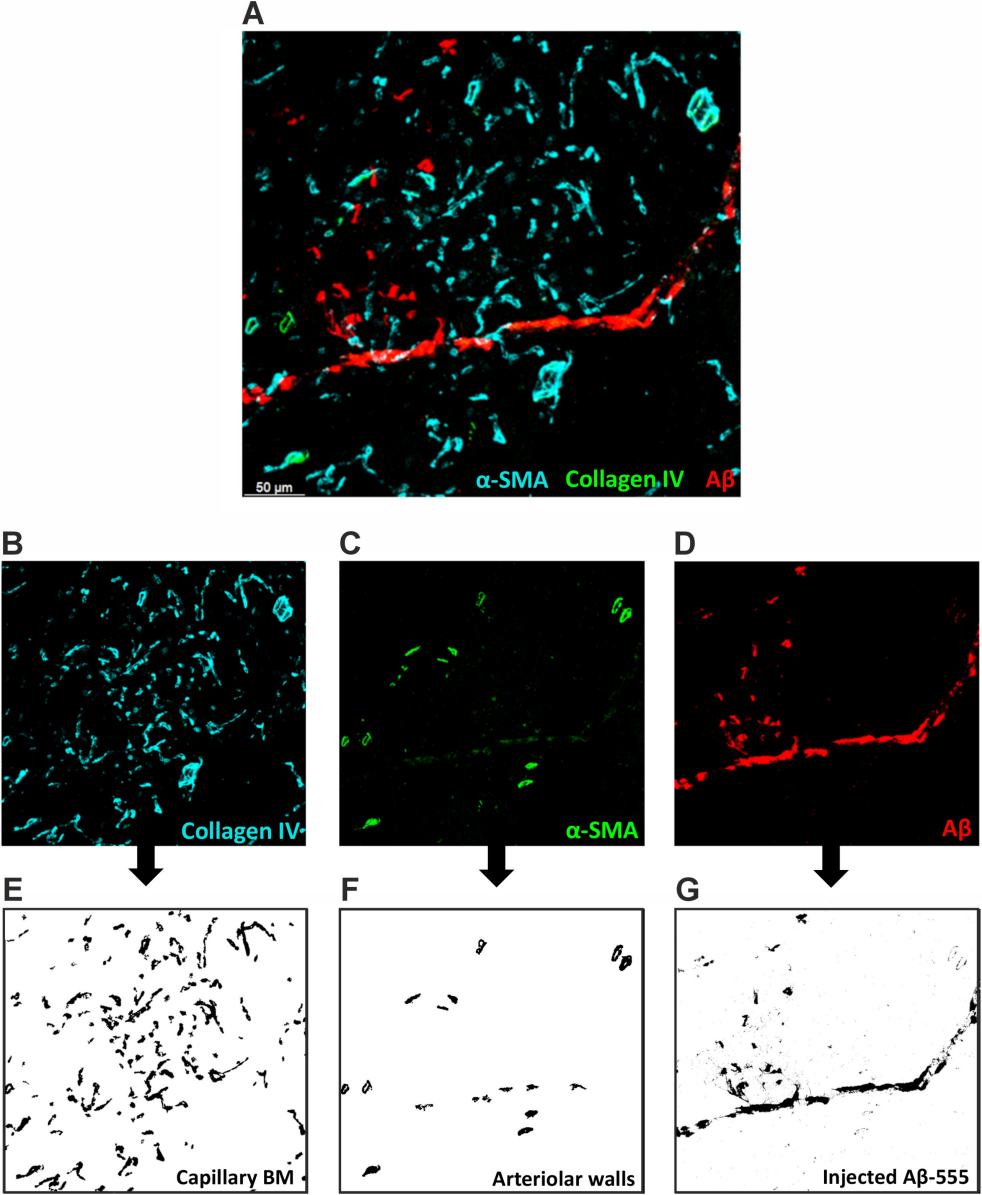
Detecting blood vessels and Aβ in mouse hippocampal brain sections using ImageJ macro. (A) Composite image. (B) Basement membrane collagen type IV. (C) Arteriole smooth muscle cell actin (α‐SMA). (D) Injected fluorescently labelled Aβ‐555. (E) Detected capillary basement membrane (BM). (F) Detected arteriolar walls. (G) Detected injected fluorescently labelled Aβ‐555.

Aβ deposits were detected using White Top Hat, Mexican‐Hat filter (https://imagej.net/ij/plugins/mexican‐hat/index.html) and intensity thresholding methods (Figure 1). Aβ load was quantified as the percentage of vessel wall occupied by Aβ fluorescence. Aβ aggregate size (in pixels) was measured within 1024 × 1024 pixel images (581.25 μm × 581.25 μm, 0.57 μm/pixel). The number of *Z* steps (≈0.69 μm each) varied per image.

### 2.4 Data Visualisation and Statistical Analysis

#### 2.4.1 Data Preprocessing

Statistical analysis and data visualisation were performed using R (Version 4.5.0) in the RStudio integrated development environment. A *p* value of less than 0.05 was considered statistically significant for all tests. For confocal microscopy data, each data point represented Aβ coverage of a single vessel (arteriole or capillary). Aβ aggregate size (in pixels) within and outside arterioles was also compared between groups. Prior to statistical analysis, two extreme outlier data points were removed from the aggregate size datasets based on a prespecified criterion of being more than 20 times larger than the next‐largest value: one aggregate from the dataset of sizes outside arterioles (size = 31,691 pixels) and one from the dataset of sizes within the arteriolar walls (size = 26,211 pixels). Additionally, one image from a control group mouse was excluded from the arteriole coverage analysis due to a technical failure of the image analysis macro, which was unable to detect any arterioles in that specific image, leading to the misclassification of deposits outside arterioles.

#### 2.4.2 Statistical Modelling

The following outcomes were compared between the Aβ + CLU group and the Aβ only control group: (1) the percentage of vessel area covered by Aβ in arterioles; (2) the percentage of vessel area covered by Aβ in capillaries; (3) the size of individual Aβ aggregates within the arteriolar walls (intramural deposits); and (4) the size of individual Aβ aggregates outside of arterioles. A Generalized Linear Mixed‐Effects Model (GLMM) approach was chosen for all outcomes. This methodology was selected because it correctly accounts for the hierarchical, nested structure of the data, where multiple aggregates or vessels are nested within each image, and multiple images are nested within each mouse. In all models, the experimental group (Aβ only control vs. Aβ + CLU) was included as a fixed effect, whereas MouseID and ImageID were included as nested random effects (1|MouseID/ImageID) to account for biological and technical variability. To ensure statistical validity, the specific distribution family for each GLMM was chosen based on the nature of the dependent variable. For vessel coverage in arterioles and capillaries, the dependent variable was proportional data that included a substantial number of true zero values. To handle this structure without artificially altering the data, a Zero‐Inflated Beta GLMM was chosen. This advanced model works in two parts: a Beta regression component to model the percentage coverage for vessels with nonzero amyloid and a logistic regression component that models the probability of a vessel having exactly zero coverage, thus preserving the important biological information within the zero values. For the analysis of aggregate size, the dependent variable was nonnegative count data (pixels) that exhibited significant skewness and overdispersion. Therefore, a GLMM with a Tweedie distribution was selected, as its flexibility provides a more accurate fit for this type of data. All models were fitted using the glmmTMB package (version 1.1.11). The initial GLMM for intramural aggregate size produced a benign convergence warning, which was investigated by fitting a simplified model; the stability of the estimates confirmed the full model was robust and its results reliable.

#### 2.4.3 Data Visualisation

Data visualisation was performed using the ggplot2 package (Version 3.5.2) and other packages from the tidyverse suite (dplyr 1.1.4, forcats 1.0.0). To directly and honestly visualise the output of the statistical models, estimated marginal means and their 95% confidence intervals were calculated using the ggeffects package (Version 2.3.0) and plotted as bar charts with error bars. Additionally, to transparently show the underlying data distributions, faceted boxplots showing the data for each of the 10 mice individually, with all individual data points overlaid, were created.

## 3 Results

Confocal microscopy image analysis was conducted on a total of 96 images obtained from 10 mice (five Aβ only Control, five Aβ + Clusterin). From these images, amyloid coverage was quantified for 689 arterioles and 1158 capillaries. Furthermore, a total of 27,237 aggregates outside arterioles and 3085 intramural aggregates were measured.

### 3.1 CLU Injection Had No Effect on Vascular Aβ Deposition

**FIGURE 2:**
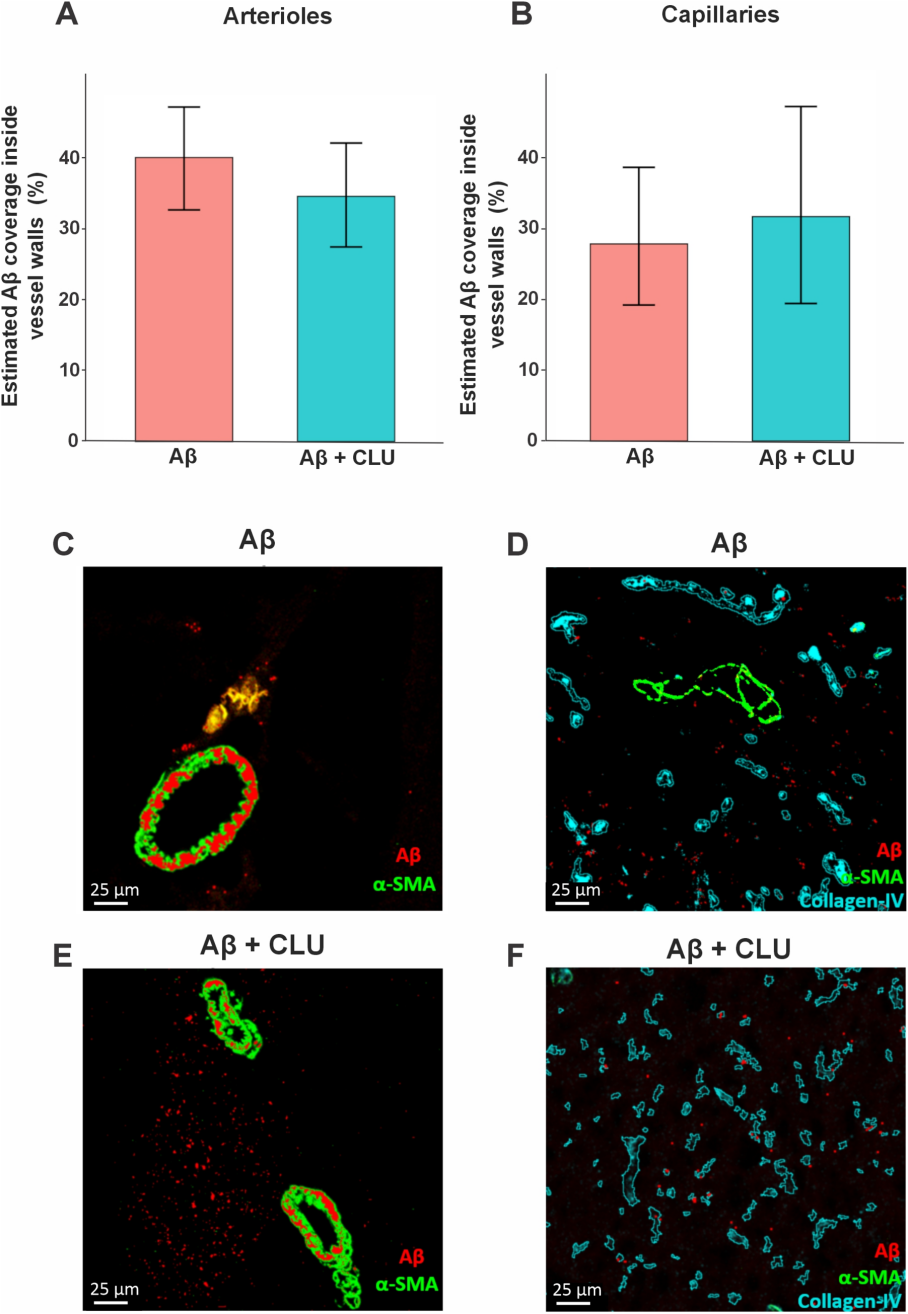
Effect of CLU on vascular Aβ coverage within arterioles and capillaries. The mean percentage of Aβ coverage within the walls of (A) arterioles and (B) capillaries. Error bars represent the 95% confidence intervals (CI). The model revealed no statistically significant difference in mean Aβ coverage for either arterioles or capillaries between the two groups. Representative confocal images of cerebral arterioles from (C) Aβ only control and (E) Aβ + CLU, identified by α‐SMA (green), with Aβ shown in red. Representative confocal images of the cerebral capillary network from (D) Aβ only control and (F) Aβ + CLU, identified by the basement membrane marker Collagen‐IV (cyan), with Aβ shown in red. Scale bar = 25 μm.

**FIGURE 3:**
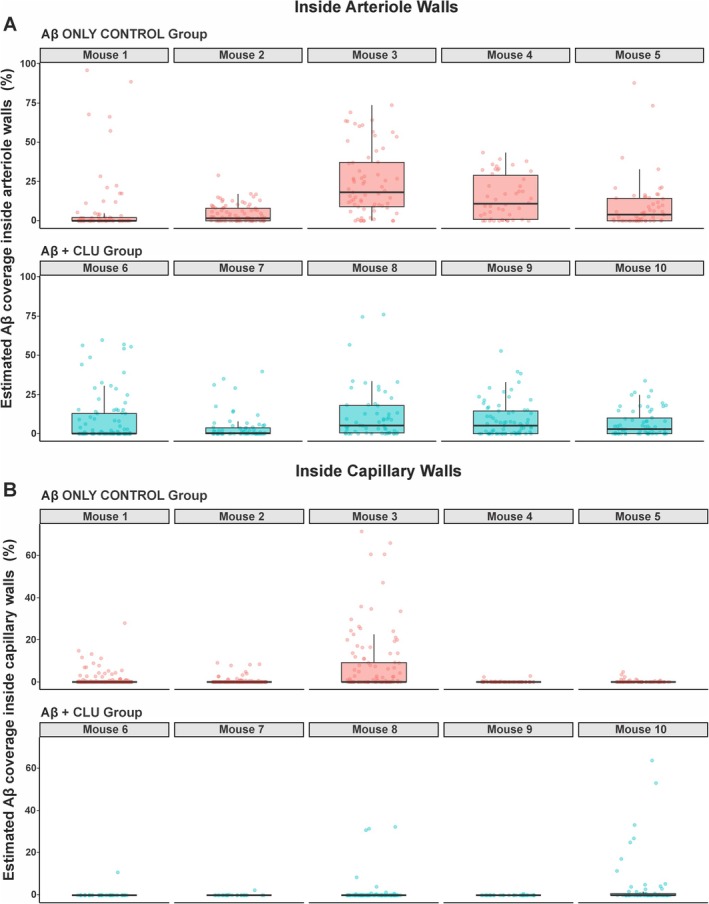
Distribution of cerebral vascular Aβ coverage for individual mice. The estimated percentage of (A) arteriolar wall area and (B) capillary wall area covered by Aβ for each of the 10 mice in the study. Each panel represents a single mouse, with the top row (pink) displaying the five Aβ only control mice (Mice 1–5) and the bottom row (blue) displaying the five Aβ + CLU mice (Mice 6–10). Within each panel, a boxplot shows the median and interquartile range of Aβ coverage for all arterioles quantified from that animal. The individual data points for each arteriole are overlaid as scattered points (jittered for visibility).

The effect of clusterin on the percentage of vessel area covered by Aβ was quantified in both arterioles and capillaries using Zero‐Inflated Beta GLMMs (Figure 2). In arterioles, the median Aβ coverage was 3.38% (IQR = 14.9%) in the Aβ only control group and 2.03% (IQR = 11.7%) in the Aβ + CLU group (Figure 2A). Statistical modelling revealed this difference was not statistically significant (estimate = −0.242, SE = 0.234, *z* = −1.033, *p* = 0.301). In capillaries, the median Aβ coverage was 0% (IQR = 0%) for both groups, and statistical modelling similarly revealed no significant effect of clusterin injection (estimate = 0.171, SE = 0.363, *z* = 0.469, *p* = 0.639) (Figure 2B). Plotting of vascular Aβ coverage of each vessel per mouse revealed the distribution of the raw data (Figure 3A,B).

### 3.2 CLU Injection Decreased Aβ Aggregate Size Within the Walls of Arterioles but Did Not Affect Aβ Aggregate Size in the Tissue Outside Arterioles

**FIGURE 4:**
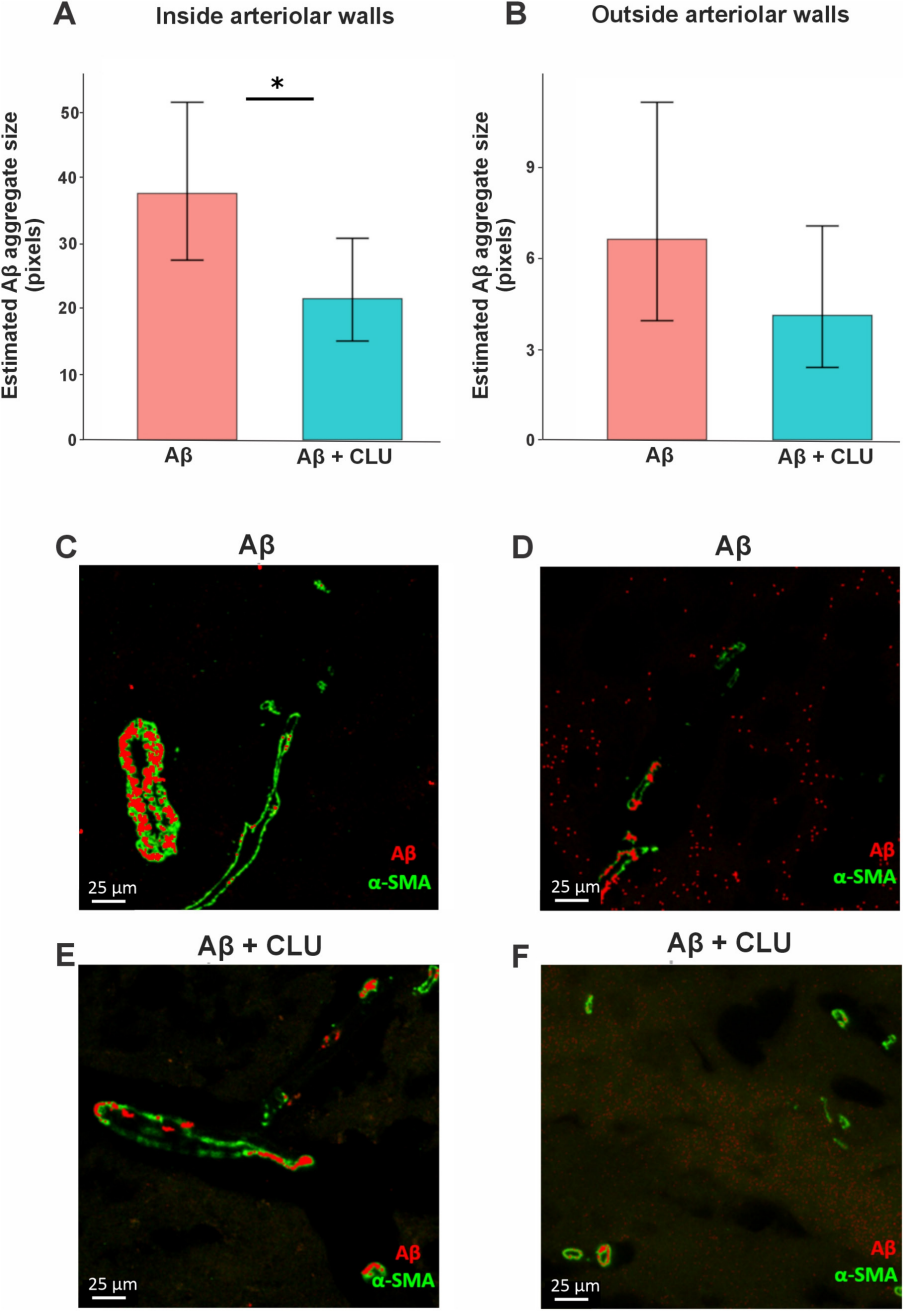
Effect of CLU on Aβ aggregate size. (A) The mean estimated Aβ aggregate size deposited within the walls of arterioles in the Aβ only control and the Aβ + CLU group. (B) The mean estimated Aβ aggregate size deposited outside the walls of arterioles in the Aβ only control and Aβ + CLU group. Error bars represent the 95% confidence intervals (CI). Representative confocal images of cerebral arterioles, identified by α‐SMA staining (green), showing intramural Aβ deposits (red) in the (C) Aβ only control and (E) Aβ + CLU group. Representative confocal images focusing on Aβ aggregates outside of the main arteriolar structures in the (D) Aβ only control and (F) Aβ + CLU group. Scale bar = 25 μm. *p < 0.05.

**FIGURE 5:**
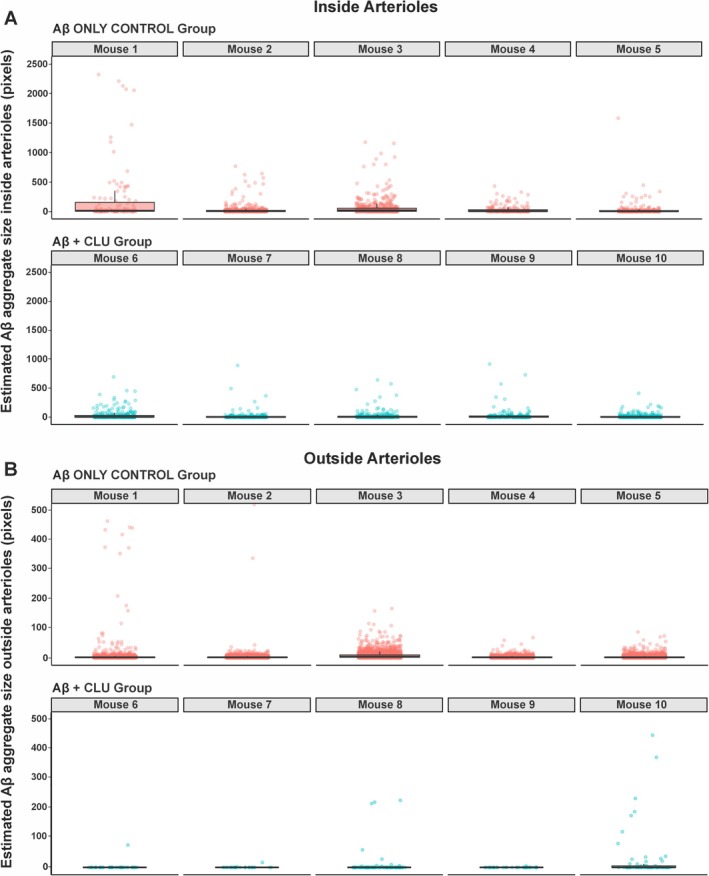
Distribution of intramural Aβ aggregate size within cerebral arterioles for individual mice. Visualisation of the raw data distribution of the estimated Aβ aggregate size deposited (A) within arteriolar walls and (B) outside arterioles for each of the 10 mice in the study. Each panel represents a single mouse, with the top row displaying the five Aβ only control mice (Mice 1–5) and the bottom row displaying the five Aβ + CLU mice (Mice 6–10). Within each panel, a boxplot shows the median and interquartile range of amyloid deposit size for all arterioles quantified from that animal. The individual data points for each arteriole are overlaid as scattered points (jittered for visibility).

We compared the size of Aβ aggregates both within and outside arterioles (Figure 4). This type of analysis was not extended to capillaries due to the unequal number of capillaries detected between the Aβ + CLU and the Aβ control groups, which may have hindered an accurate discrimination between vascular and nonvascular brain tissue. Aβ + CLU injection led to a decrease in Aβ aggregate size within the walls of arterioles (*p* = 0.02) (GLMMs with a Tweedie distribution; Figure 4A). Although the median aggregate size was similar between the control group (8 pixels) and the Aβ + CLU group (7 pixels), the GLMM, which accounts for the full data distribution, estimated that aggregates in the Aβ + CLU group were significantly smaller (estimate = −0.557, SE = 0.243, *z* = −2.295). For Aβ aggregates outside arterioles, there was no statistically significant difference in size between the Aβ control group (median = 2 pixels, IQR = 3) and the Aβ + CLU group (median = 2 pixels, IQR = 4), as determined by the GLMM (estimate = −0.477, SE = 0.383, *z* = −1.246, *p* = 0.213) (Figure 4B). Plotting the size of each Aβ aggregate deposited within and outside of arterioles for each mouse revealed the distribution of the raw data (Figure 5A,B).

## 4 Discussion

It has previously been shown that the accumulation of insoluble Aβ‐40 and Aβ‐42 in cerebral vessels of APP23 mice is accompanied by increased clusterin expression, supporting the observation that clusterin levels are significantly elevated in brains from AD patients compared to controls and correlate with regional Aβ deposition [36, 37]. However, to date, studies have been unable to consistently define whether the role of clusterin in relation to Aβ aggregation, deposition and toxicity in the brain is protective or pathogenic. In this study, we investigated the pattern of Aβ deposition in hippocampal cerebral vessels of clusterin knockout mice, following intrahippocampal injection of fluorescent Aβ alone or in combination with human recombinant clusterin. We demonstrate that the size of fluorescent Aβ deposits in the walls of arterioles was significantly reduced in the Aβ + CLU group compared to the Aβ only control group, whereas no difference in Aβ deposit size was observed in the tissue outside arterioles. Interestingly, we observed no difference in the amount of Aβ deposited inside arterioles or capillaries between the Aβ + CLU group compared to the Aβ only control group.

Consistent with our findings, a previous study showed that intravenous administration of clusterin or reconstituted high‐density lipoprotein particles containing clusterin in the APP23 rodent model of cerebral amyloidosis (prior to the endogenous clusterin elevation observed in this model) significantly lowered the number of CAA‐affected arteries [37]. Furthermore, immunohistochemistry of brain tissue in these APP23 mice found that the deposition and size of Aβ deposits within the parenchyma did not differ significantly in the presence of systemic clusterin [37]. Wojtas et al. [30] previously demonstrated that exogenously added CLU reduced binding of Aβ_40_ and Aβ_42_ to isolated cerebral vessels in an ex vivo mouse model, suggesting that clusterin reduces Aβ binding to vessel walls. However, as we did not observe any difference in overall Aβ load within arteriole walls, it is not possible to conclude whether the presence of CLU directly influences Aβ binding to vessel walls. The ability of clusterin to inhibit Aβ oligomerisation supports our finding that in the presence of CLU, Aβ aggregate size decreases within arteriole walls, and it may be that the generation of these smaller, more numerous aggregates within vessels could explain why total Aβ coverage remained constant whereas the Aβ oligomerisation state was altered in the presence of CLU.

Our study showed that the presence of CLU did not alter Aβ deposit size outside arterioles. This could be due to the fact that clusterin's ability to influence Aβ aggregation is more efficient within vessels, either as a result of reduced penetration of recombinant clusterin into the parenchyma compared to the vessels or the vascular milieu providing a better environment for CLU's chaperone function. Specifically, the interaction of clusterin with vascular basement membranes and pericytes has been shown to be critical in the protein's Aβ chaperone function [38, 39]. It is also important to note that vascular Aβ deposits are typically rich in Aβ‐40 isoform, whereas parenchymal plaques are usually composed of Aβ‐42. In this study, Aβ‐40 was injected intrahippocampally, and therefore, this could influence Aβ deposition patterns between the vasculature and parenchyma. There is no evidence of clusterin being present in the walls of veins; thus, glymphatic clearance of Aβ is unlikely in this case, but it may be that the complexes of Aβ‐CLU are eliminated via other mechanisms apart from IPad. The binding of CLU to Aβ could in fact result in the diverting of Aβ towards the LRP2‐mediated transport of stable Aβ‐CLU complexes into the circulation [30].

Clusterin is capable of binding to Aβ oligomers of varying sizes, from dimers to 50‐mers [21]. It is possible that smaller CLU‐Aβ complexes can more easily enter and be cleared along perivascular spaces, whereas larger complexes remain outside vessels awaiting alternative clearance routes, such as LRP‐2 mediated transport across the BBB (blood–brain barrier). This could explain why, in the presence of clusterin, Aβ aggregate size within vessel walls was significantly smaller compared to outside vessels. Although our study focused on a single concentration of exogenous CLU and Aβ 1–40, it has also previously been shown that in the presence of increasing concentrations of exogenous human recombinant CLU and Aβ, the deposition of Aβ in vessel walls decreased, even at high Aβ concentrations, supporting our findings that the absence of CLU facilitates the aggregation of Aβ [30]. It is worth noting that the findings from this study represent a single time point of 10 min postinjection. Future studies incorporating additional timepoints may be able to further interrogate the impact of clusterin on Aβ clearance along perivascular drainage routes and should continue to focus on defining the specific mechanisms mediating these observed changes in Aβ deposition in the presence/absence of clusterin.

Previous studies have relied upon the use of transgenic AD mouse models to accumulate Aβ levels such that Aβ deposition in cerebral vessels can be assessed in the presence or absence of clusterin [37]. However, the increased expression of inflammatory cytokines and microglial activation (amongst other pathological changes) in many of these models, including APP/PS1, makes it challenging to tease apart the direct effect of clusterin on Aβ clearance. Therefore, the intrahippocampal injection of clusterin knockout mice with human Aβ alone or in the presence of CLU is an important experiment to better understand how CLU modifies Aβ deposition within cerebral arterioles and a first step to investigate whether the application of recombinant clusterin could be a potential therapeutic to improve Aβ clearance and CAA pathology.

## 5 Conclusion

Taken together, our findings, alongside previous studies, suggest a critical role for clusterin in regulating the aggregation of Aβ, particularly along Intramural Peri‐Arterial routes. The decrease in Aβ deposit size in cerebral arteriole walls in the presence of clusterin is consistent with a chaperone function for clusterin, whereby its effective binding to Aβ inhibits amyloid oligomerization, enabling soluble Aβ to drain more effectively along IPAD pathways. Alternatively, the absence of the chaperone function for clusterin could impair transvascular clearance of Aβ via the endocytic receptor LRP2, subsequently redirecting Aβ along Peri‐Arterial drainage routes. There is a distinct possibility that CLU may stabilise Aβ in the extracellular spaces as plaques before it reaches its vascular/perivascular routes of clearance. Because the size and shape of plaques in the extracellular spaces of the brain do not correlate with the degree of cognitive decline [40], overall, our results suggest that clusterin facilitates the clearance of Aβ along the walls of cerebral arteries and therefore may contribute to in preventing CAA, which is a key pathological feature of AD [41].

## Author Contributions

Conceptualization: R.O.C., A.L., T.‐G.B., J.D.F., and A.V. Formal analysis: A.L. and T.‐G.B. Funding acquisition: R.O.C. Methodology: B.A.C., M.R.W., A.L., A.D.I., and T.‐G.B. Supervision: R.O.C. Genotyping: C.B. Immunofluorescence: L.C. Confocal imaging: D.M., J.M.D., and T.‐G.B. Writing – original draft: R.O.C., J.M.D., A.L., T.‐G.B., J.D.F., and M.R.W. Writing – review and editing: R.O.C., J.M.D., A.L., T.‐G.B., J.D.F., A.V., and M.R.W.

## Ethics Statement

All the procedures on mice were approved by the Veterinary Health and Food Safety Institution of Romania and in compliance with the guidelines established by the Public Health Service Guide for the Care and Use of Laboratory Animals (Ethical Approval 59/2023).

## Conflicts of Interest

The authors declare no conflicts of interest.

## Data Availability

The datasets used and/or analysed during the current study are available from the corresponding author on reasonable request.
